# A semi-nonparametric Poisson regression model for analyzing motor vehicle crash data

**DOI:** 10.1371/journal.pone.0197338

**Published:** 2018-05-23

**Authors:** Xin Ye, Ke Wang, Yajie Zou, Dominique Lord

**Affiliations:** 1 Key Laboratory of Road and Traffic Engineering of Ministry of Education, College of Transportation Engineering, Tongji University, Shanghai, China; 2 Zachry Department of Civil Engineering, Texas A&M University 3136 TAMU, College Station, TX, United States of America; Beihang University, CHINA

## Abstract

This paper develops a semi-nonparametric Poisson regression model to analyze motor vehicle crash frequency data collected from rural multilane highway segments in California, US. Motor vehicle crash frequency on rural highway is a topic of interest in the area of transportation safety due to higher driving speeds and the resultant severity level. Unlike the traditional Negative Binomial (NB) model, the semi-nonparametric Poisson regression model can accommodate an unobserved heterogeneity following a highly flexible semi-nonparametric (SNP) distribution. Simulation experiments are conducted to demonstrate that the SNP distribution can well mimic a large family of distributions, including normal distributions, log-gamma distributions, bimodal and trimodal distributions. Empirical estimation results show that such flexibility offered by the SNP distribution can greatly improve model precision and the overall goodness-of-fit. The semi-nonparametric distribution can provide a better understanding of crash data structure through its ability to capture potential multimodality in the distribution of unobserved heterogeneity. When estimated coefficients in empirical models are compared, SNP and NB models are found to have a substantially different coefficient for the dummy variable indicating the lane width. The SNP model with better statistical performance suggests that the NB model overestimates the effect of lane width on crash frequency reduction by 83.1%.

## 1. Introduction

Statistical regression models are typically used in analyzing the likelihood and severity of vehicle crashes. Recent review studies [1–4] have summarized the innovative models for examining the impact of factors (e.g., traffic, roadway and vehicle characteristics, etc.) on the likelihood of a crash and its resulting injury severity. Regarding observed crash counts, previous studies often found that some crash data are likely to demonstrate heterogeneity. This heterogeneity in the crash data can be explained as the unknown variation of the impact of explanatory variables on crash. As discussed by Mannering, et al.[3], when the crash-related information is collected, some factors affecting the likelihood and severity of the vehicle crash may not be available to the transportation safety analysts (e.g., driver’s weight, height, roadway lighting type, etc.). And different kinds of data have been applied to the safety analysis[5,6]. To date, various models have been introduced to or developed for crash modeling analysis. For example, mixed-Poisson models[7–11], latent class/Markov switching models[12–17], random parameter models[18–27].

Among these crash modeling methods, the most frequently used statistical method for modeling crash count data is the Negative Binomial (NB, also known as Poisson-gamma) model. The NB model has its inadequacy when describing certain types of crash data. The distribution assumed in the probabilistic error term related to the mean of the Poisson variable can be restrictive in terms of its ability to account for different types of heterogeneity across observations. In econometric literature, the semi-nonparametric (SNP) distribution has been introduced [28]. The SNP distribution is developed based on a squared K^th^-order polynomial expansion which can provide a smooth estimation of the distribution of the error term[29–33]. Previous studies [34–36] have shown the flexibility of SNP distribution. Thus, the SNP distribution can be used to model the probabilistic error term regarding the mean of the Poisson variable by transportation safety analysts to analyze crash data with heterogeneity.

Due to the importance of the error term related to the mean of the Poisson variable in transportation crash modeling, the objective of this study is to examine whether or not the Poisson-SNP distribution can capture the heterogeneity characteristics of crash data. To achieve this objective, crash data sets are simulated using different combinations of fixed regression parameters describing the mean and dispersion levels. Based on the simulated datasets, the parameter and distribution of the error term are estimated and compared to the true values. The simulation analysis are conducted due to the following reason: when real crash data are analyzed, the true values of regression parameters and the distribution of the error term are seldom known in practice. In contrast, in a simulation, it is possible to generate crash data with known regression parameters and an assumed distribution for error term. The simulation analysis have been adopted in some previous transportation safety studies[7,11] to evaluate the performance of different estimators. To complement outputs from simulation studies, crash data collected in California of USA are also used to compare the estimation results between the Poisson-SNP model and NB model.

## 2. Modeling methodology

This section presents the modeling methodology adopted in this paper. First, the Poisson regression model is presented using the log-gamma heterogeneity (i.e., the Negative Binomial regression model). Although the focus of this paper is to develop a Poisson regression model for crash frequency with unobserved heterogeneity following a semi-nonparametric (SNP) distribution, it will be insightful to present this state-of-practice model for comparison.

### 2.1. Negative binomial (NB) regression model: Poisson regression model with log-gamma heterogeneity

Count data models are most suited to modeling dependent variable y_*i*_ that constitutes a frequency or “count.” The dependent variable can only take non-negative integer values. In this paper, y_*i*_ represents crash frequency for road section *i*. The expectation of y_*i*_ is assumed to be *λ*_*i*_ and the count data model formulation is as follows:

$$\ln\left(\lambda_{i}\right)=x_{i}\beta +\varepsilon_{i},$$ (1)

where *x*_*i*_ is a vector of explanatory variables indicating characteristics for road section *i*; *β* is a vector of coefficients associated with *x*_*i*_. *ε*_*i*_ is a random variable representing heterogeneity that accounts for unobserved factors and other random disturbances. Since y_*i*_ constitutes count data, the probability of y_*i*_ conditional on *ε*_*i*_ is given as:

$$\mathrm{Pr}\left(y_{i}|\varepsilon_{i}\right)=\frac{\exp\left(-\lambda_{i}\right)\;{\lambda_{i}}^{y_{i}}}{y_{i}!}.$$ (2)

The Negative Binomial (NB) regression model is formulated based on the assumption that exp(*ε*_*i*_) = *t*_*i*_ follows a gamma distribution, denoted as Γ(1/*α*^2^,*α*^2^). The corresponding probability density function is:

$$f(t_{i})=\frac{{t_{i}}^{1/\alpha^{2}-1}}{{\left(\alpha^{2}\right)}^{1/\alpha^{2}}\Gamma (1/\alpha^{2})}\exp\left(-\frac{t_{i}}{\alpha^{2}}\right),t_{i}>0,$$ (3)

$$\mathrm{where}\;\Gamma (z)=\int_{0}^{\infty }t^{z-1}e^{-t}dt.$$ (4)

The expectation and standard deviation of *t* are equal to 1 and *α*, respectively. By integrating *t*_*i*_ over its distributional domain, one may obtain the unconditional probability of y_*i*_ as:

$$\mathrm{Pr}\left(y_{i}\right)=\int_{-\infty }^{\infty }Pr(y_{i}|t_{i})\;f(t_{i})\;dt_{i}=\frac{\Gamma (1/\alpha^{2}+y_{i})}{\Gamma (1+y_{i})\Gamma (1/\alpha^{2})}{r_{i}}^{y_{i}}{\left(1-r_{i}\right)}^{1/\alpha^{2}},$$ (5)

$$\mathrm{where}\;r_{i}=\frac{\alpha^{2}exp(x_{i}\beta )}{\alpha^{2}\exp\left(x_{i}\beta \right)+1}.$$ (6)

Cameron and Trivedi [37] proposed this unconditional probability function with a closed-form solution. This formulation has allowed the NB model to be widely applied for modeling count data in many different areas, including transportation.

It is to be noted that the true heterogeneity in the model is not *t*_*i*_, but *ε*_*i*_, which accounts for the presence of unobserved variables or factors excluded from the vector *x*_*i*_. Since *ε*_*i*_ is equal to ln(*t*_*i*_), the underlying distributional assumption on *ε*_*i*_ is the log-gamma distribution and the probability density function can be derived as:

$$f(\varepsilon_{i})=\frac{1}{\Gamma (1/\alpha^{2})}exp\{\frac{1}{\alpha^{2}}[\varepsilon_{i}-\ln\left(\alpha^{2}\right)]-e^{\left[\varepsilon_{i}-\ln\left(\alpha^{2}\right)\right]}\},-\infty <\varepsilon_{i}<+\infty .$$ (7)

It is not a symmetric function with respect to the variable *ε*_*i*_, indicating that the distribution of the random variable *ε*_*i*_ is asymmetric in nature[38].

### 2.2. Poisson regression with SNP heterogeneity

To improve the flexibility of the distribution for unobserved heterogeneity, one may choose to use the SNP distribution for representing heterogeneity *ε*_*i*_. The probability density function for the SNP distribution is usually specified as:

$$f(\varepsilon )=\frac{{\left(\sum_{m=0}^{K}a_{m}\varepsilon^{m}\right)}^{2}\varphi (\varepsilon )}{\int_{-\infty }^{+\infty }{\left(\sum_{m=0}^{K}a_{m}\varepsilon^{m}\right)}^{2}\varphi (\varepsilon )d\varepsilon }.$$ (8)

In Eq (8), "K" is the length of the polynomial, "m" is an index increasing from 0 to "K", a_m_ is a constant coefficient, and φ(ε) represents the probability density function (PDF) of the standard normal distribution. The denominator ensures that $\int_{-\infty }^{+\infty }f(\varepsilon )d\varepsilon =1$. The denominator in Eq (8) can be extended and written in the following form, where "n" is another index increasing from 0 to "K":

$$\int_{-\infty }^{+\infty }{\left(\sum_{m=0}^{K}a_{m}\varepsilon^{m}\right)}^{2}\varphi (\varepsilon )d\varepsilon =\sum_{m=0}^{K}\sum_{n=0}^{K}a_{m}a_{n}\int_{-\infty }^{+\infty }\varepsilon^{m+n}\varphi (\varepsilon )d\varepsilon$$ (9)

$\int_{-\infty }^{+\infty }\varepsilon^{m+n}\varphi (\varepsilon )d\varepsilon$ in Eq (9) is actually the expectation of ε^*m*+*n*^, which can be calculated based on the moment-generating function and derived recursion formulae.

$$\mathrm{Define}\;I(n)=\int_{-\infty }^{+\infty }\varepsilon^{n}\varphi (\varepsilon )d\varepsilon ,\;\mathrm{then},\;I(0)=1,\;I(1)=0\;\mathrm{and}\;I(n)=(n-1)\;I(n-2),\;\mathrm{when}\;n\geq 2.$$ (10)

$$\mathrm{Thus},\;\mathrm{the}\;\mathrm{denominator}\;\int_{-\infty }^{+\infty }{\left(\sum_{m=0}^{K}a_{m}\varepsilon^{m}\right)}^{2}\varphi (\varepsilon )d\varepsilon =\sum_{m=0}^{K}\sum_{n=0}^{K}a_{m}a_{n}I(m+n).$$ (11)

Under the assumption of the SNP distribution, one can integrate *ε*_*i*_ over its distributional domain and obtain the unconditional probability of y_*i*_ as:

$$\mathrm{Pr}\left(y_{i}\right)=\int_{-\infty }^{+\infty }\mathrm{Pr}\left(y|\varepsilon_{i}\right)f\left(\varepsilon_{i}\right)d\varepsilon_{i}=\int_{-\infty }^{+\infty }\left\{\frac{\exp\left[-\exp\left(x_{i}\beta +\varepsilon_{i}\right)\right]{\left[\exp\left(x_{i}\beta +\varepsilon_{i}\right)\right]}^{y_{i}}}{y_{i}!}\cdot \frac{{\left(\sum_{m=0}^{K}a_{m}\varepsilon_{i}{}^{m}\right)}^{2}\varphi \left(\varepsilon_{i}\right)}{\sum_{m=0}^{K}\sum_{n=0}^{K}a_{m}a_{n}I\left(m+n\right)}\right\}\;d\varepsilon_{i}$$ (12)

The key difference in comparison to the NB regression model is that the unconditional probability function presented in Eq (12) does not have a closed-form solution. The numerical method of Gauss–Hermite quadrature is applied to approximate the unconditional probability as follows:

$$Pr(y_{i})\approx \sum_{j=1}^{J}\left\{w_{j}\{\frac{\exp\left[-exp(x_{i}\beta +s_{j})\right]{\left[exp(x_{i}\beta +s_{j})\right]}^{y_{i}}}{y_{i}!}\}∙[\frac{{\left(\sum_{m=0}^{K}a_{m}{s_{j}}^{m}\right)}^{2}\varphi (s_{j})}{\sum_{m=0}^{K}\sum_{n=0}^{K}a_{m}a_{n}I(m+n)}]\right\}$$ (13)

Gaussian quadrature[39] is a sophisticated procedure that can accurately evaluate the integrals in the likelihood function with a small number (usually 10–20) of supporting points. In this study, 30 supporting points are applied to ensure a high level of accuracy for integral evaluations. The values of nodes and weights of Gaussian-Hermit quadrature are listed in Table 1 for interested readers.

**Table 1:** Node and weight values in Gauss–Hermite quadrature (30 supporting points).

**j**	**1**	**2**	**3**	**4**	**5**
**s**	-6.86335	-6.13828	-5.53315	-4.98892	-4.48306
**w**	0.834247	0.649098	0.569403	0.522526	0.491058
**j**	**6**	**7**	**8**	**9**	**10**
**s**	-4.00391	-3.54444	-3.09997	-2.66713	-2.24339
**w**	0.468375	0.451321	0.438177	0.427918	0.419895
**j**	**11**	**12**	**13**	**14**	**15**
**s**	-1.82674	-1.41553	-1.00834	-0.60392	-0.20113
**w**	0.413679	0.408982	0.405605	0.40342	0.402346
**j**	**16**	**17**	**18**	**19**	**20**
**s**	0.201129	0.603921	1.00834	1.41553	1.82674
**w**	0.402346	0.40342	0.405605	0.408982	0.413679
**j**	**21**	**22**	**23**	**24**	**25**
**s**	2.24339	2.66713	3.09997	3.54444	4.00391
**w**	0.419895	0.427918	0.438177	0.451321	0.468375
**j**	**26**	**27**	**28**	**29**	**30**
**s**	4.48306	4.98892	5.53315	6.13828	6.86335
**w**	0.491058	0.522526	0.569403	0.649098	0.834247

The log-likelihood function over the sample consisting of “N” observations can be formulated as:

$$LL(\beta ,a)=\sum_{i=1}^{N}ln\{\sum_{j=}^{J}\left\{w_{j}\{\frac{\exp\left[-exp(x_{i}\beta +s_{j})\right]{\left[exp(x_{i}\beta +s_{j})\right]}^{y_{i}}}{y_{i}!}\}∙[\frac{{\left(\sum_{m=0}^{K}a_{m}{s_{j}}^{m}\right)}^{2}\varphi (s_{j})}{\sum_{m=0}^{K}\sum_{n=0}^{K}a_{m}a_{n}I(m+n)}]\right\}\}.$$ (14)

The standard Maximum Likelihood Estimation (MLE) method can be applied to estimate unknown parameters in the vectors “*β”* and “*a”* by maximizing the log-likelihood function in Eq (14). The model estimation is an exploratory procedure, in which the polynomial length “K” needs to start from “1” and then gradually increases to involve more coefficients into the vector “a”. The likelihood ratio test can be applied to examine whether adding more coefficients can significantly improve the goodness-of-fit of model. The model estimation results will be finalized when adding more coefficients fails to significantly improve the goodness-of-fit (GOF) measure.

## 3. Simulation experiments

In this section, a number of simulation experiments are conducted to demonstrate the capability of the SNP distribution to approximate different types of distributions for unobserved heterogeneities in Poisson regression models. Those distributions include log-gamma distributions, normal distributions, a bimodal distribution and a trimodal distribution.

### 3.1 SNP model approximating NB model (Poisson model with log-gamma heterogeneity)

The NB model is the most practical modeling approach for crash frequency. It will be insightful to examine whether an SNP distribution can well approximate the log-gamma heterogeneity in NB models. The simulation experiments are designed as below:

λ=exp(1.0−0.3∙x1+0.4∙x2+ε),

where “x_1_” and “x_2_” independently follow a uniform distribution between 0 and 5; “*ε*” represents the unobserved heterogeneity following the log-gamma distribution, whose PDF is given in Eq (7) and the parameter *α*^2^ = 0.8. Then, the count variable “y” is drawn from a Poisson distribution associated with the parameter λ.

The sample size is setup at 1000. Based on the random sample consisting of the dependent variable “y” and explanatory variables “x_1_” and “x_2_”, an NB model can be estimated and shown in the left part of Table 2. As expected, the model coefficients are highly consistent with their true values. With the same sample, an SNP model can be estimated as well and the estimation results are presented in the right part of Table 2 for comparison. It should be noted that the coefficient a_0_ needs to be fixed at 1 for identification. The high flexibility of the SNP distribution causes that the intercept in a regression model and the expectation of “*ε*” may not be simultaneously identifiable. To solve this issue and facilitate comparisons, the intercept of the SNP model is fixed at the value of intercept in the NB model.

**Table 2:** Comparison between NB and SNP Models (*α*^2^ = 0.8, Sample Size = 1000).

	NB Model		SNP Model	
Variable (True Value)	Value	SE	Value	SE
b_0_ (1.0)	1.0031	0.0908	1.0031	—
b_1_ (-0.3)	-0.2969	0.0239	-0.2969	0.0232
b_2_ (0.4)	0.3829	0.0243	0.3864	0.0244
*α*^2^(0.8)	0.8113	0.0541	—	—
a_0_	—	—	1.0000	—
a_1_	—	—	-0.0581	0.0692
a_2_	—	—	-0.1393	0.0388
a_3_	—	—	-0.0521	0.0135
a_4_	—	—	0.0207	0.0065
LL(β)	**-2372.46**		**-2372.61**	

**Fig 1:**
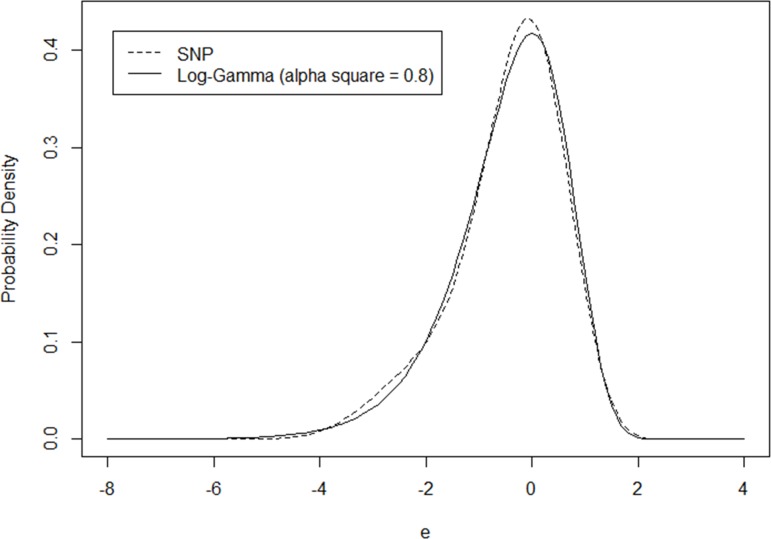
Comparison of SNP and Log-Gamma distributions (*α*^2^ = 0.8).

As shown, when the length of polynomial (i.e. the “K” value) reaches 4, the SNP model almost perfectly replicates the NB model results, including the log-likelihood value at convergence, model coefficients, and the plot of heterogeneity distribution (as in Fig 1).

**Fig 2:**
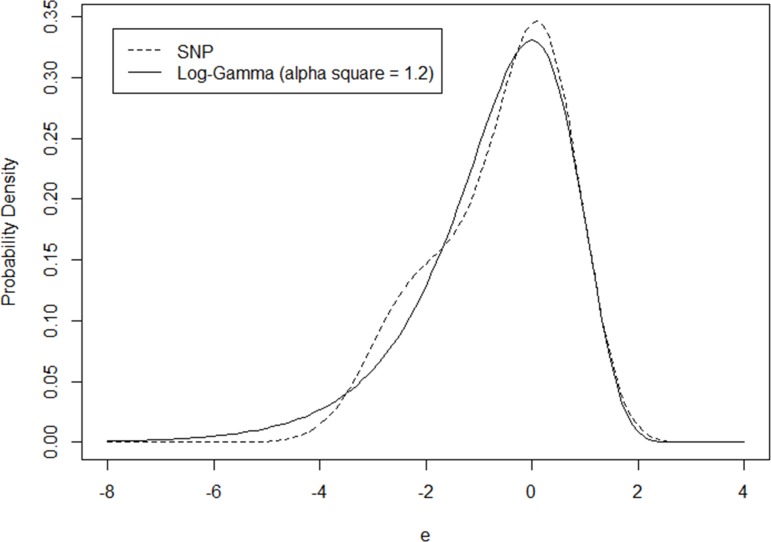
Comparison of SNP and Log-Gamma distributions (*α*^2^ = 1.2).

Table 3 provides similar comparisons between NB and SNP models when ***α***^**2**^ takes the value of 1.2. In this case, the approximation is not as good as before. However, the relative difference between model coefficients is still less than 3% while the log-likelihood values at convergence and the plots of heterogeneity distributions are close to each other (as in Fig 2).

**Table 3:** Comparison between NB and SNP Models (*α*^2^ = 1.2, Sample Size = 1000).

	NB Model		SNP Model	
Variable (True Value)	Value	SE	Value	SE
b_0_ (1.0)	1.0157	0.1047	1.0157	—
b_1_ (-0.3)	-0.3450	0.0279	-0.3537	0.0264
b_2_ (0.4)	0.4007	0.0276	0.3915	0.0169
*α*^2^(1.2)	1.2215	0.0760	—	—
a_0_	—	—	1.0000	—
a_1_	—	—	0.0496	0.0572
a_2_	—	—	-0.0459	0.0450
a_3_	—	—	-0.0895	0.0131
a_4_	—	—	0.0213	0.0070
LL(β)	**-2358.37**		**-2359.19**	

### 3.2 SNP model approximating Poisson model with normal heterogeneities

In this subsection, the SNP distribution is applied to approximate normal heterogeneities in Poisson regression models. The simulation experiments are designed as below:

λ=exp(−0.3∙x1+0.4∙x2+ε),

where “x_1_” and “x_2_” still follow independently uniform distribution between 0 and 5; “*ε*” follows a normal distribution and $PDF(\varepsilon )=\frac{1}{\sigma \sqrt{2\pi }}exp\left[-\frac{{\left(\varepsilon -\mu \right)}^{2}}{2\sigma^{2}}\right]$, where *μ* = 0 and *σ* = 0.8 or 1.2; the count variable “y” is drawn from a Poisson distribution associated with the parameter λ.

**Fig 3:**
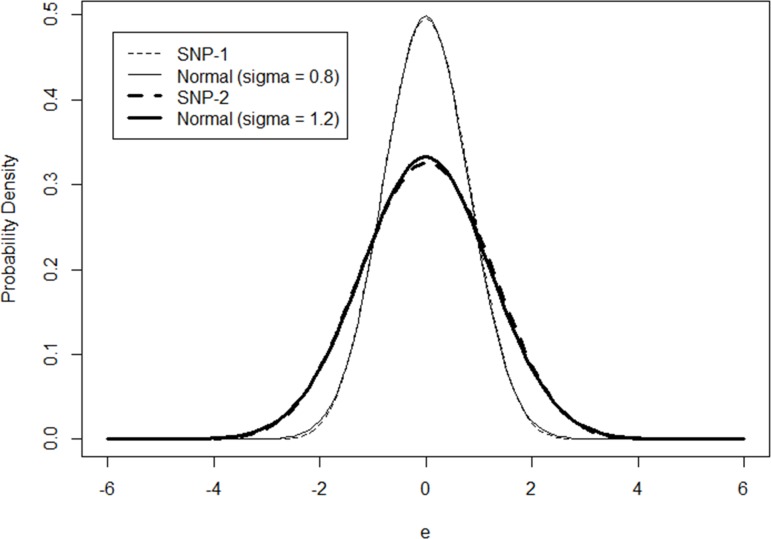
Comparison of SNP and normal distributions (*σ* = 0.8 or 1.2).

The sample size is also setup at 1000. Based on the random sample consisting of the dependent variable “y” and explanatory variables “x_1_” and “x_2_”, SNP models can be estimated to approximate the normal heterogeneities in the Poisson regression model. The model estimation results are presented in Table 4. With the polynomial length of 2, SNP models can almost perfectly approximate the normal distributions when “*σ*” takes the value of 0.8 or 1.2. The model coefficients are highly consistent with their true values and differences between the exact and simulated heterogeneity distributions are almost invisible (as in Fig 3).

**Table 4:** SNP models to approximate normal heterogeneities.

	SNP Model 1 (*σ* = 0.08)		SNP Model 2 (*σ* = 1.2)	
Variable (True Value)	Value	SE	Value	SE
b_1_ (-0.3)	-0.2993	0.0113	-0.3042	0.0151
b_2_ (0.4)	0.4090	0.0039	0.3946	0.0045
a_0_	1.0000	—	1.0000	—
a_1_	0.0059	0.0332	0.0067	0.0305
a_2_	-0.1194	0.0185	0.0980	0.0218
LL(β)	-2112.09		-2462.97	

(Sample Size = 1000)

### 3.3 SNP model approximating bimodal and trimodal distributions

This subsection further exhibits the great flexibility of the SNP distribution to approximate a bimodal distribution and a trimodal distribution. The simulation experiments are designed as below:

λ=exp(−0.3∙x1+0.4∙x2+ε),

where “x_1_” and “x_2_” still follow independently uniform distribution between 0 and 5. The unobserved heterogeneity ε = 3 ∙ D(u_1_ > 0.4) + 1.5 ∙ u_2_ + 0.5 ∙ η − 2.5, where D () is an indicator function, “𝜂” follows the standard normal distribution while “u_1_” and “u_2_” independently follow the standard uniform distribution. The sample size is setup at 500. Since it is challenging to derive the analytical PDF of the mixture distribution for the random variable “*ε*”, Kernel Density Estimation (KDE) approach is employed to estimate density for each e_i_ in the arithmetic sequence (e_i_ = -6.0, -5.9, … 5.9, 6.0) based on the random sample and following equation:

$$f_{KDE}(e_{i})=\sum_{j=1}^{500}K_{h}(e_{i}-\varepsilon_{j})/500.$$ (15)

**Fig 4:**
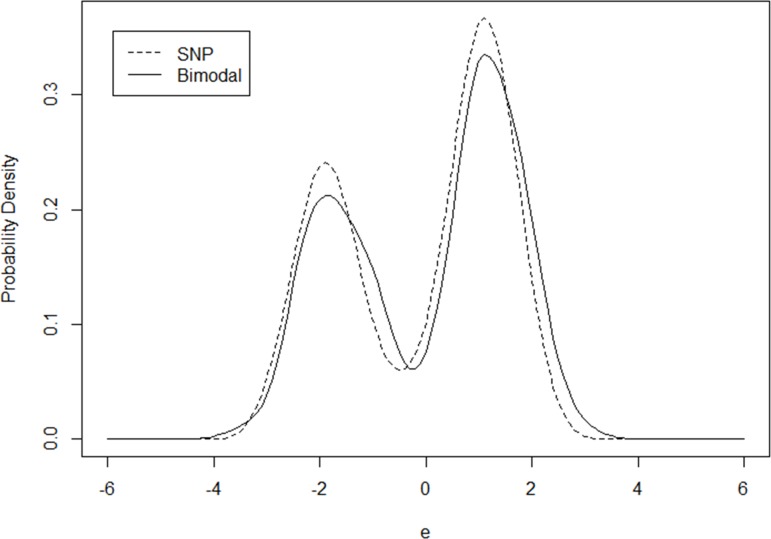
Comparison of SNP and bimodal distributions.

In the formula, K_h_(u) = ϕ(u/h)/h. Namely, the PDF of standard normal distribution is chosen as the smooth function K_h_(u) and the bandwidth “h” is setup at 0.3. The estimated probability density is plotted as a solid curve in Fig 4. As shown, it is a typical bimodal distribution with two explicit modal points.

After the vector “λ” is generated, the count variable “y” is drawn from a Poisson distribution based on this vector. Then, an SNP model is estimated to approximate the bimodal distribution and the estimation results are provided in the left part of Table 5. When “K” reaches 5, the model coefficients are close to their true values and the SNP distribution can mimic the bimodal distribution reasonably well, which is plotted as a dashed curve in Fig 4.

**Table 5:** SNP models to approximate bimodal and trimodal heterogeneities.

	SNP Model 1 (Bimodal Distribution)		SNP Model 2 (Trimodal Distribution)	
Variable (True Value)	Value	SE	Value	SE
b_1_ (-0.3)	-0.2801	0.0119	-0.2804	0.0061
b_2_ (0.4)	0.4139	0.0051	0.4107	0.0021
a_0_	1.0000	—	1.0000	—
a_1_	0.8984	0.2587	-0.0496	0.0942
a_2_	0.9218	0.3554	-0.2594	0.0712
a_3_	-0.3543	0.1535	-0.0160	0.0514
a_4_	-0.0637	0.0485	0.0804	0.0088
a_5_	0.0174	0.0170	-0.0007	0.0047
LL(β)	-1289.46		-1327.89	

Sample Size = 500

**Fig 5:**
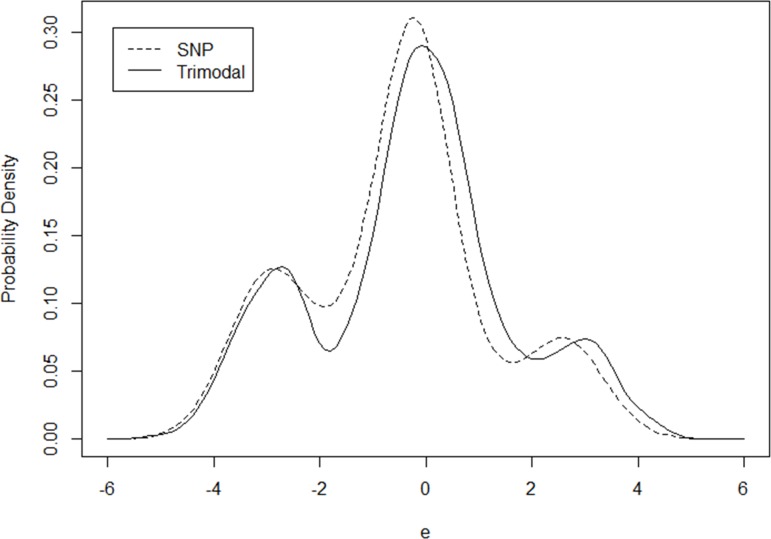
Comparison of SNP and trimodal distributions.

A last experiment is conducted to mimic a trimodal distribution. The unobserved heterogeneity *ε* = 3 D(u_1_ > 0.8)– 3 D(u_2_ > 0.7) + 2 u_3_ + 0.5 𝜂 – 1.0, where “𝜂” follows the standard normal distribution while “u_1_”, “u_2_” and “u_3_” independently follow the standard uniform distribution. The KDE approach is applied to estimate kernel densities using a bandwidth of 0.4 for better smoothness. The estimated distribution is then plotted as a solid curve in Fig 5. The model coefficients, which are close to their true values, are presented in the right part of Table 5. The dashed line in Fig 5 represents the SNP distribution mimicking the trimodal distribution. As shown, the SNP distribution correctly exhibits the feature of the trimodal distribution with three modal points and mimics the overall distribution reasonably well.

In summary, the simulation experiments demonstrate the strong capability of the SNP distribution to approximate different types of distributions (e.g. unimodal, bimodal and trimodal distributions) for unobserved heterogeneity in Poisson regression models. In terms of the performance, the SNP distribution can almost perfectly approximate a symmetric unimodal distribution like normal distribution, well approximate a skewed unimodal distribution like log-gamma distribution and reasonably approximate bimodal and trimodal distributions. With consideration of heterogeneity following the SNP distribution, all the model coefficients are highly consistent or fairly close to their true values. Consequently, it should be appropriate to apply the flexible SNP distribution to explore potential problems, such as non-symmetricity, skewness or multimodality, etc., in the distribution of the unobserved heterogeneities within a Poisson regression model.

## 4. Data description

An empirical crash dataset is used to demonstrate the capability of SNP distribution in modeling unobserved heterogeneities. The crash observations were collected on 1443 rural highway sections in California State of USA from 1993 to 2002. This dataset contains sufficient explanatory variables, which can be used to develop a well-defined mean functional form for NB and SNP models. Table 6 provides the summary statistics of variables for the California data. The mean and variance of observed crash frequencies are 15.6 and 1973.9 (the maximum number of crashes is 1192), respectively. Thus, the variance to mean ratio is 126.5. During the 10-year period, 22522 crashes occurred on 1334 out of the 1443 road sections (92.4%).

**Table 6:** Summary statistics of variables for the California data.

Variable	Minimum	Maximum	Mean	Std. Dev.
Number of crashes (10 years)	0.00	**1192.00**	**15.61**	44.43
Segment length (in miles) (L)	0.10	4.37	0.50	0.52
Average daily traffic over 10 years (AADT)	1372.00	78300.00	16001.57	13088.46
Ln(L·10)	0.00	3.78	1.26	0.79
Ln(AADT)	7.22	11.27	9.39	0.77
Median width (in feet)	0.00	99.00	34.56	32.34
Lane width (in feet)	6.00	15.00	12.01	0.39
Right shoulder width (in feet)	0.00	23.00	7.85	2.80

## 5. Empirical estimation results

This section presents the comparison results between the NB and SNP models. Table 7 presents all the estimate results and overall performance measurements of both NB model and SNP model of crash frequency for comparisons. In the SNP model, the log-likelihood value at convergence can be gradually improved until the polynomial length “K” reaches 3. The performance measurements are listed at the bottom of the table, including the log-likelihood value at convergence [i.e. LL(β)], Deviance, Akaike information criterion (AIC) and Bayesian information criterion (BIC). The following formulae are used to compute those performance measurements:

Deviance=−2∙LL(β),

AIC=2[k−LL(β)],BIC=ln(n)∙k−2∙LL(β),

where “n” represents the sample size and “k” represents the number of parameters estimated in the model. A greater value in LL(β) or a less value in Deviance indicates a better goodness-of-fit (GOF) for the data.

**Table 7:** Crash frequency model estimation results.

	NB Model		SNP Model	
Variable	Value	SE	Value	SE
Intercept	-7.0561	0.6873	-7.0561	—.
Ln[10×length]	1.0000	—	1.0000	—.
Ln(AADT)	1.0711	0.0267	1.0046	0.0187
Median width (ft) / 10	-0.0348	0.0083	-0.0369	0.0056
Lane width (ft)	**-0.1266**	0.0542	**-0.0677**	0.0171
Right shoulder width (ft)	-0.0733	0.0093	-0.0699	0.0043
α^2^	0.5035	0.0239	—	—
a_0_	—	—	1.0000	—
a_1_	—	—	-0.3242	0.0336
a_2_	—	—	-0.1714	0.0164
a_3_	—	—	0.0408	0.0093
Overall Performance Measurements				
Sample size	1443		1443	
LL(β)	-4480.06		**-4441.44**	
Deviance	8960.13		**8882.87**	
AIC	8972.13		**8896.87**	
BIC	9003.78		**8933.79**	

As shown in Table 7, the SNP model greatly improves the GOF for the data relative to the NB model after 3 additional parameters for the SNP distribution are specified into the model. AIC and BIC are two alternative criteria for model selection by penalizing the number of parameters in models and avoiding overfitting issues. A smaller value of AIC or BIC indicates a better performance of the SNP model than that of the NB model. It implies that it is worth specifying additional coefficients to better describe the distribution of the unobserved heterogeneity and further improve the model performance. In addition, the Chi-squared test is applied to examine whether adding more coefficients can significantly improve the goodness-of-fit of the SNP model. When the polynomial length “K” reaches 3, the Chi-squared test value is 164.36 relative to the log-likelihood value with “K” at 1 and the critical value is 5.99 for 2 degrees of freedom. Since the increase of the polynomial length fails to further significantly improve the goodness-of-fit, the model is finalized at the polynomial length of 3.

**Fig 6:**
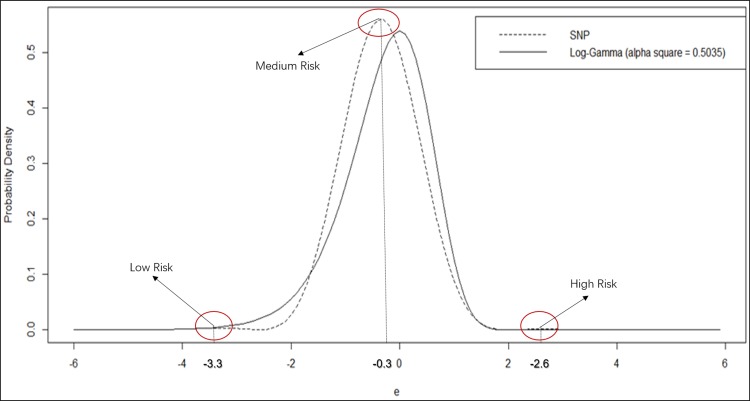
Comparison of SNP and Log-Gamma distributions in crash frequency models.

Fig 6 visualizes the SNP distribution and compares it with the estimated log-gamma distribution in the NB model. It is interesting to see that the estimated SNP distribution exhibits three visible modal points, although the left and right ones are fairly minor. They are presumed to correspond to three groups of observations in the sample. The major one occurs near -0.3 on the coordinate of “*ε*” and takes a density value of 0.56, corresponding to the largest group in the middle of the distributional domain. This group consists of almost all the (about 99.5%) observations in the sample. The left mode occurs near -3.3 on the coordinate and the relevant small group consists of about 0.4% of all the observations. The right modal point occurs near +2.6 on the coordinate and the relevant group only consists of 0.1% of observations. Since the heterogeneity “*ε*” represents unobserved or unspecified factors affecting crash frequency, those results indicate the existence of three groups of highway segments exposed to different levels of crash risk, which may be denoted as “low risk”, “medium risk” and “high risk” groups. About 6 (= 1443∙0.4%) highway segments from the sample fall into the “low risk” group. If the expected crash frequencies are compared between the “low risk” and “medium risk” groups, the expectation of “low risk” can be only 5% of that of “medium risk” [i.e. exp(−3.3 + 0.3)] even if all the observed and specified factors are the same. Similarly, there are only about 1 ~ 2 (≈1443·0.1%) highway segments falling into the “high risk” group, where the expected crash frequency can be more than 18 times [i.e. exp(2.6 + 0.3)] as much as that of “medium risk” group when all the observed and specified factors are the same.

However, the details revealed by the SNP distribution are ignored by the log-gamma distribution assumed in the NB model. If comparing two distributions, one may envision that the log-gamma distribution has already been extended to represent both left and middle groups. Unfortunately, the log-gamma distribution is a unimodal distribution and therefore cannot exhibit more than one mode to well represent a multimodal distribution. On the other hand, the log-gamma distribution is a skewed distribution in nature, which cannot well reflect a more symmetric error distribution of observations in the middle group. As a result, the GOF of the SNP model is much better than that of the NB model thanks to its advantages to have multiple modes and represent a more symmetric distribution.

In addition to the overall model performance, the SNP model brings great benefit to improve the precision of model estimators. If comparing the standard errors of coefficient estimators between SNP and NB models, one may see that some of them are reduced by a few times. As we know, MLE estimators are consistent and efficient only if the distributional assumption is valid. When the heterogeneity is well mimicked by the SNP distribution, the model coefficient estimators have much less standard errors and are more precise than those in the NB model based on the inappropriate unimodal and skewed distribution for unobserved heterogeneity. When comparing magnitude of estimated coefficients, one may find that SNP and NB models have similar coefficient estimators except that of the dummy variable indicating the lane width. The SNP model with better statistical performance suggests that the NB model substantially overestimates the effect of lane width on crash frequency reduction by 83.1% ({1 − exp(−0.1266)} v.s.{1 − exp(−0.0677)}). The striking difference is probably caused by a better representation of the error distribution in the SNP model.

## 6. Conclusions and discussions

In this paper, the authors specify a semi-nonparametric (SNP) distribution to represent the unobserved heterogeneity in a Poisson regression model for crash frequency analysis. Relative to the unimodal log-gamma distribution in the conventional negative binomial model, the SNP distribution is highly flexible to mimic different types of distributions. When the length of polynomial increases, the SNP distribution can approximate a large family of distributions, including symmetric or asymmetric unimodal distribution and different types of multimodal distributions. Traffic crash analysts can take advantage of its flexibility to release distributional restrictions imposed by the conventional modeling method and explore the most appropriate distributional form for the unobserved heterogeneity.

In the empirical study based on the crash dataset collected from the California State of USA, the SNP distribution classifies the observations from the sample into three groups, which are exposed to different levels of risk. The SNP model fits data substantially better than the conventional NB model and provides more precise model coefficient estimators. The NB model is found to substantially overestimate the effect of lane width on crash frequency reduction relative to the SNP model based on more robust estimation of unobserved heterogeneity.

Future research may be carried out in the following three directions. At first, an approach may be required to classify observations into the groups identified by the SNP model. With this approach, there may be great potential to identify “high-risk” and “low-risk” locations associated with unobserved risk factors for further considerations. In addition, the crash model may be re-estimated based on the observations belonging to the “medium-risk” group, where the unobserved heterogeneity is more narrowly distributed. If it can be realized, the goodness-of-fit of the model may be further improved, while all the model coefficients will reflect the situation with the most “medium-risk” locations since “outliers” in “high-risk” and “low-risk” locations are omitted from the sample. Second, the SNP model needs to be applied to some other crash frequency datasets to further examine its applicability in different occasions. Third, there are different methods to capture the heterogeneity. Instead of modifying the distribution of the random component *ε*_*i*_, a random parameter model can also be explored to capture the heterogeneity and improve the goodness-of-fit of model. In future research, a random parameter model may be developed and compared with the SNP model and the traditional NB model.

## Supporting information

S1 Data Data. (SAV)

S1 Fig Comparison of SNP and Log-Gamma distributions (*α*^2^ = 0.8). (TIF)

S2 Fig Comparison of SNP and Log-Gamma distributions (*α*^2^ = 1.2). (TIF)

S3 Fig Comparison of SNP and normal distributions (*σ* = 0.8 or 1.2). (TIF)

S4 Fig Comparison of SNP and bimodal distributions. (TIF)

S5 Fig Comparison of SNP and trimodal distributions. (TIF)

S6 Fig Comparison of SNP and Log-Gamma distributions in crash frequency models. (TIF)

S1 Table Node and weight values in Gauss–Hermite quadrature (30 supporting points). (TIF)

S2 Table Comparison between NB and SNP models (*α*^2^ = 0.8, Sample Size = 1000). (TIF)

S3 Table Comparison between NB and SNP models (*α*^2^ = 1.2, Sample Size = 1000). (TIF)

S4 Table SNP models to approximate normal heterogeneities (Sample Size = 1000). (TIF)

S5 Table SNP models to approximate bimodal and trimodal heterogeneities (Sample Size = 500). (TIF)

S6 Table Summary statistics of variables for the California data. (TIF)

S7 Table Crash frequency model estimation results. (TIF)
