# Supplementary figures and images for: A semi-nonparametric Poisson regression model for analyzing motor vehicle crash data
Source: PLoS One. 2018 May 23;13(5):e0197338. doi: 10.1371/journal.pone.0197338 (PMC5965849; doi:10.1371/journal.pone.0197338)

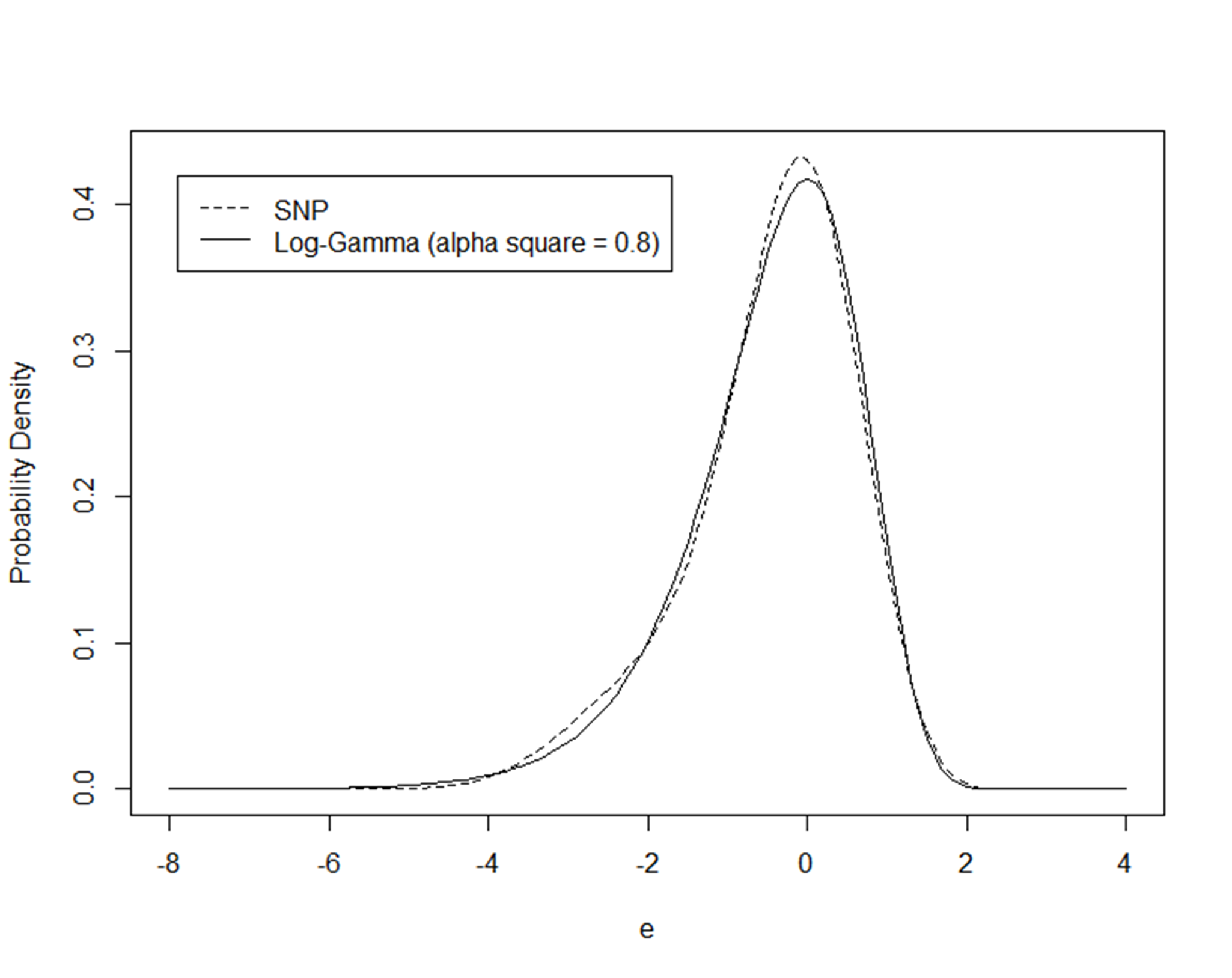

Supplement: S1 Fig — (TIF) [file pone.0197338.s002.tif]

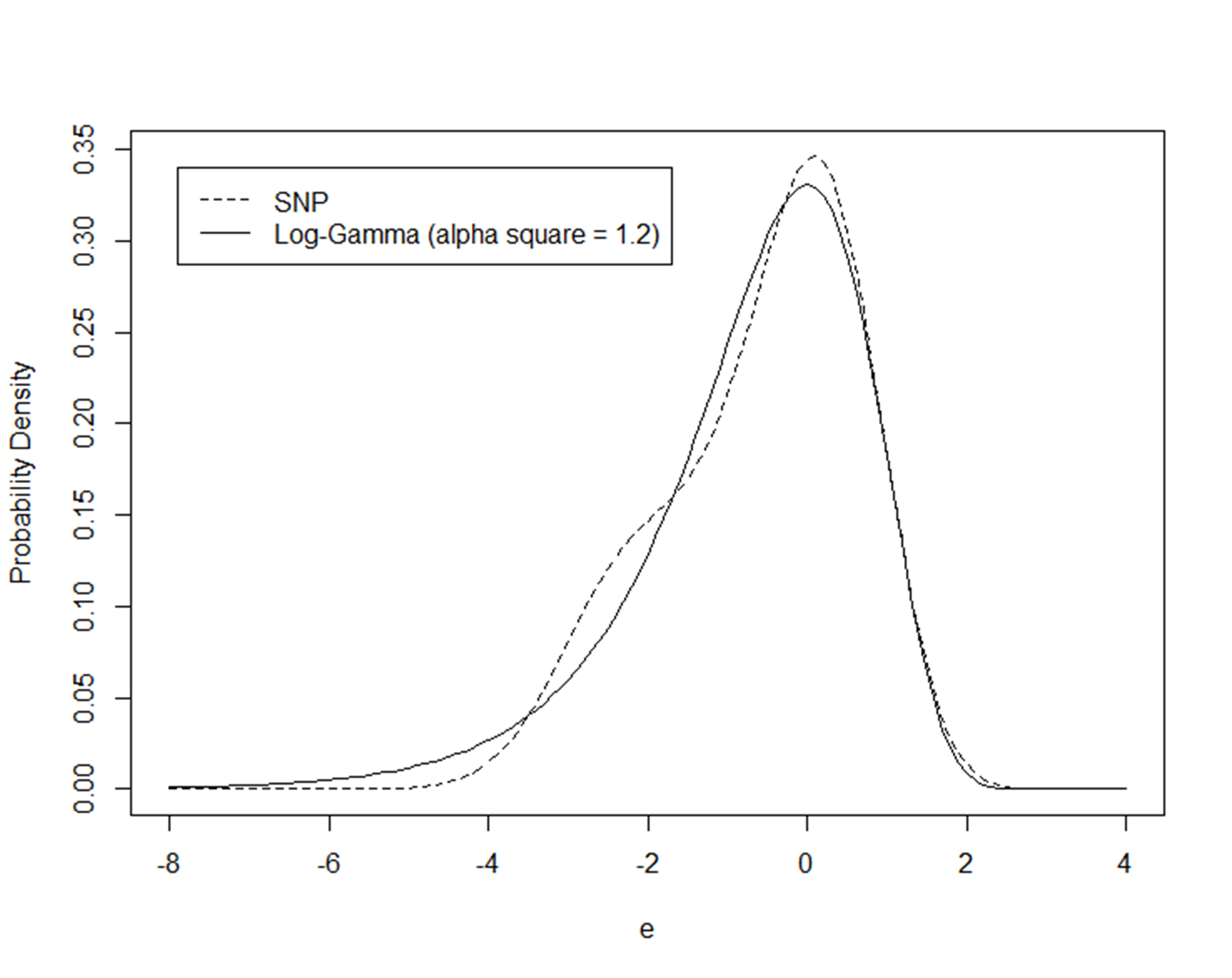

Supplement: S2 Fig — (TIF) [file pone.0197338.s003.tif]

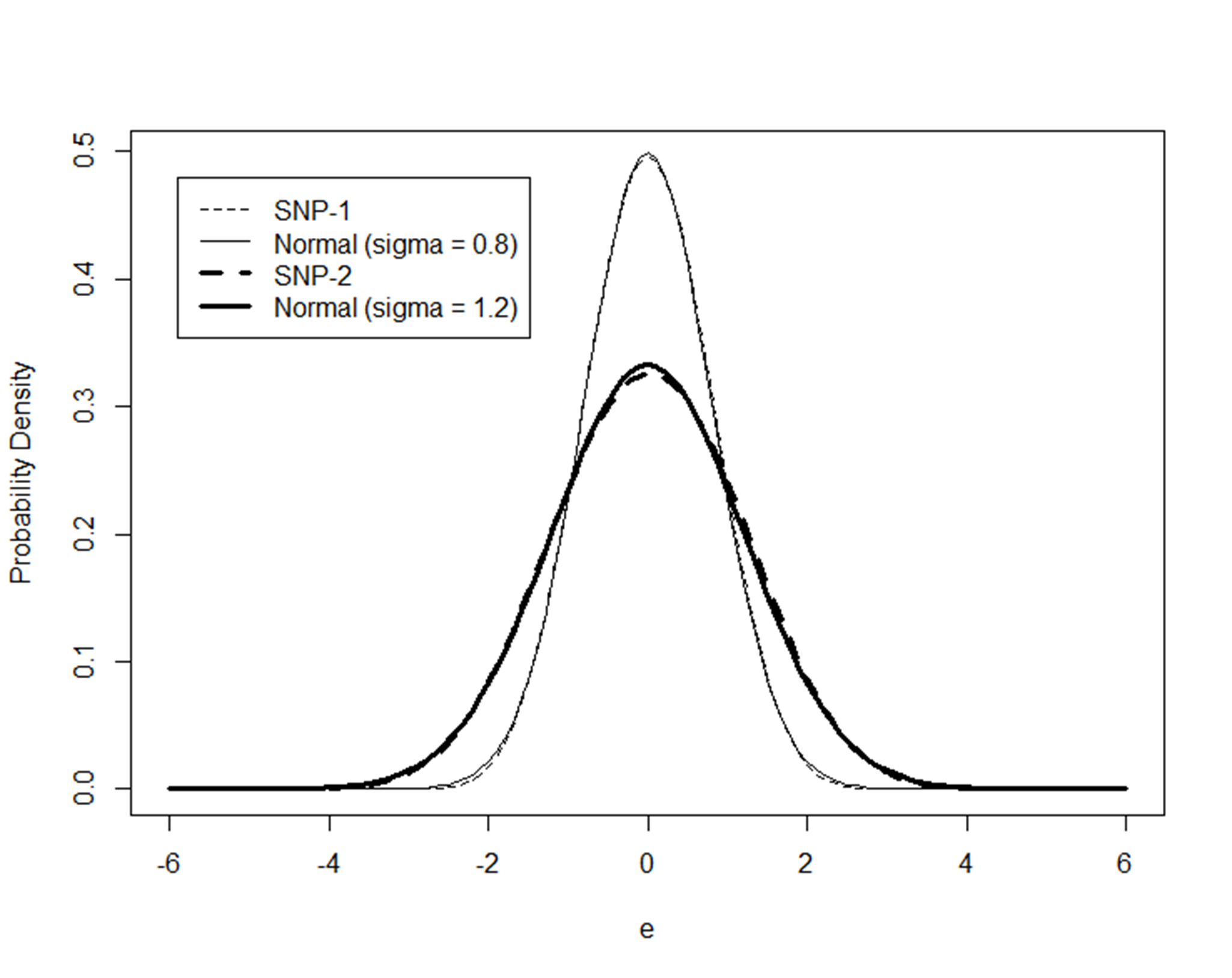

Supplement: S3 Fig — (TIF) [file pone.0197338.s004.tif]

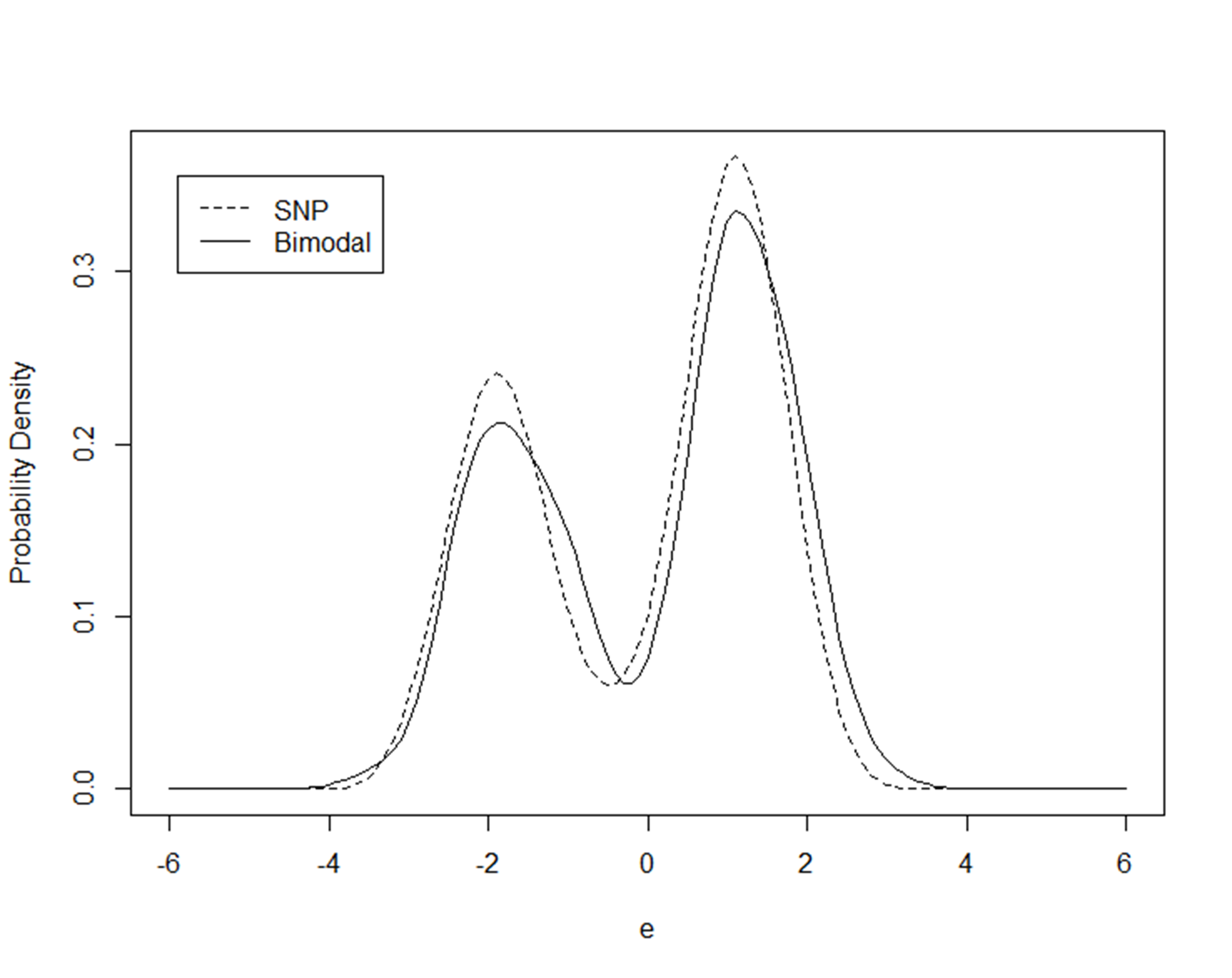

Supplement: S4 Fig — (TIF) [file pone.0197338.s005.tif]

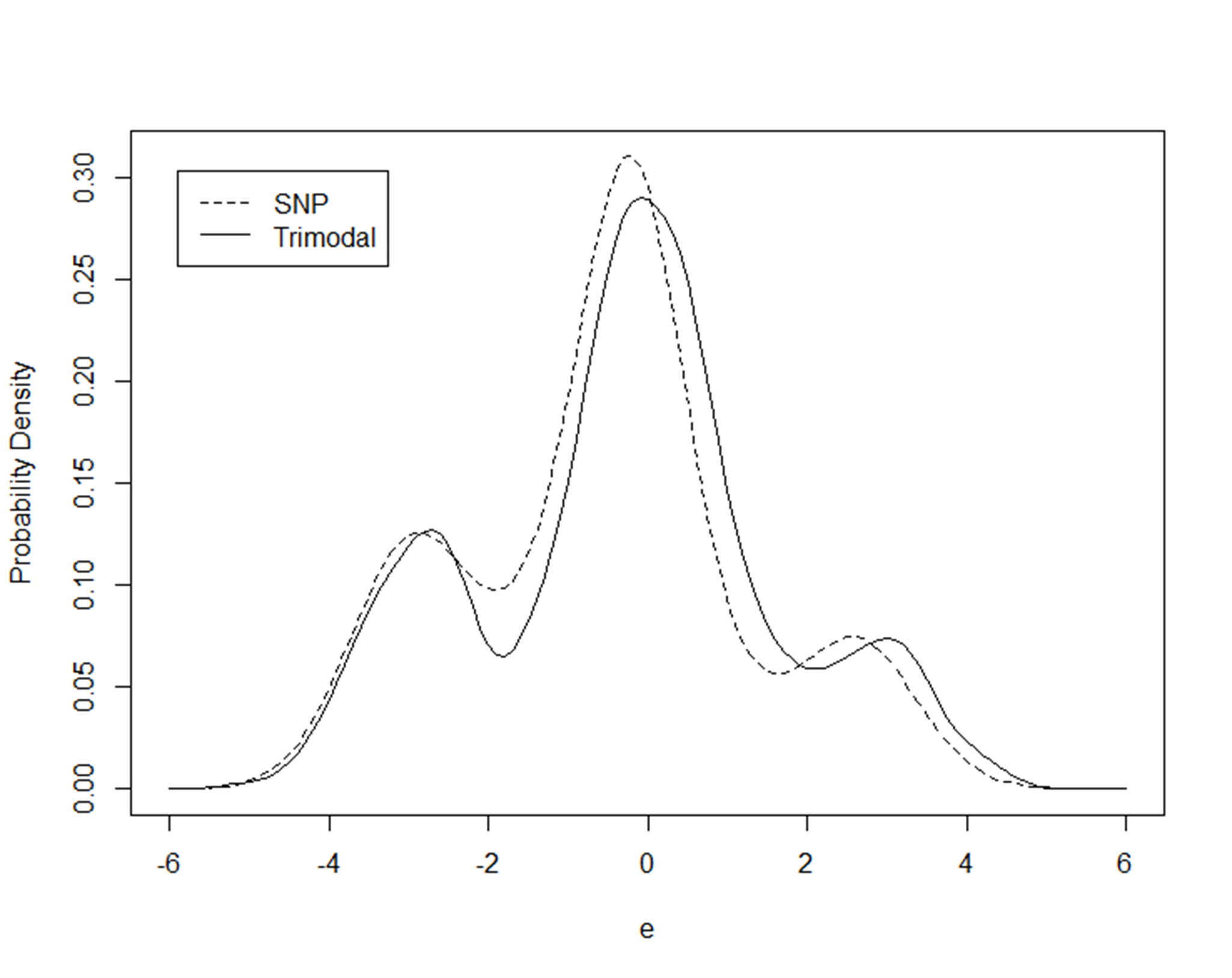

Supplement: S5 Fig — (TIF) [file pone.0197338.s006.tif]

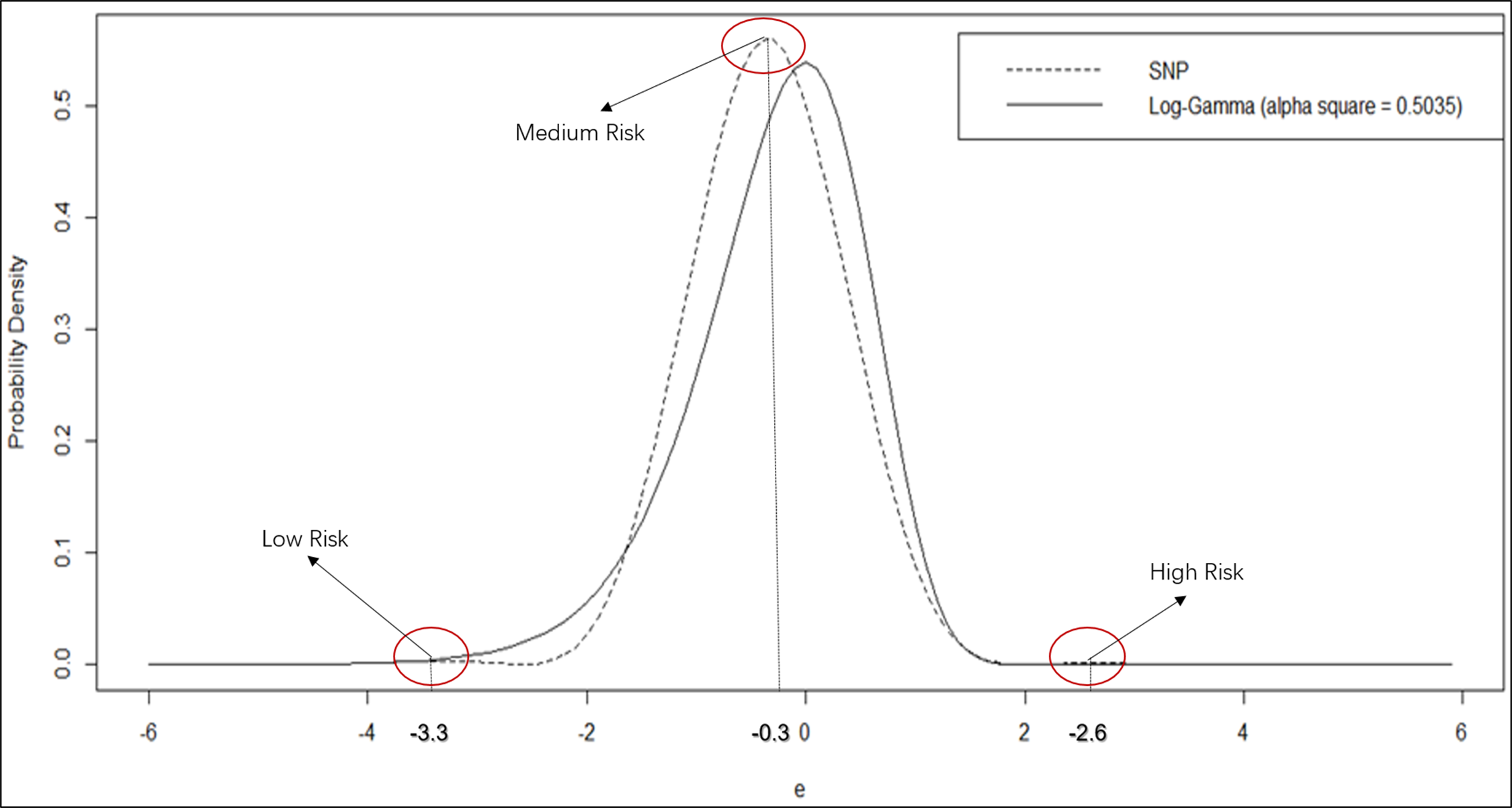

Supplement: S6 Fig — (TIF) [file pone.0197338.s007.tif]

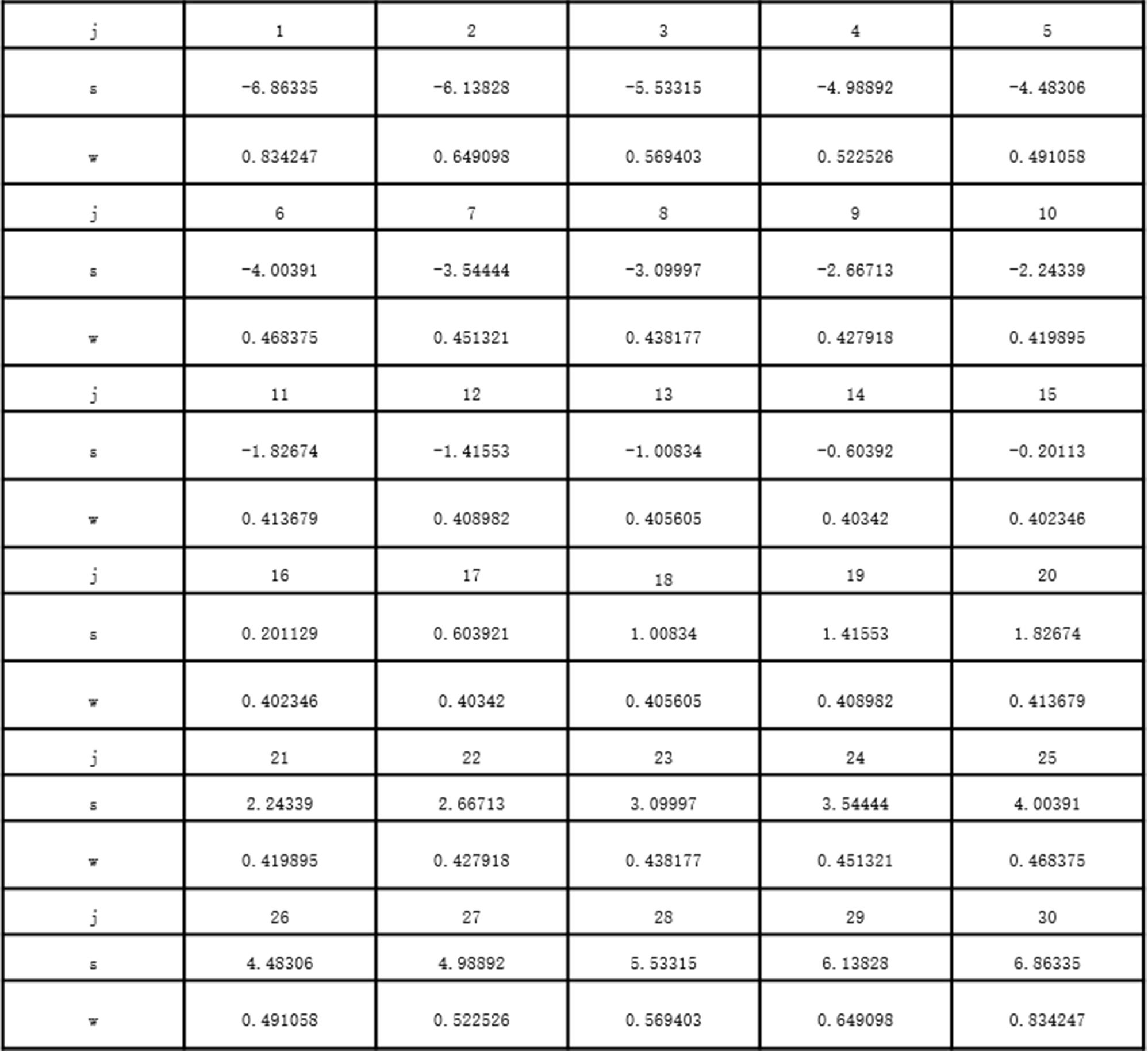

Supplement: S1 Table — (TIF) [file pone.0197338.s008.tif]

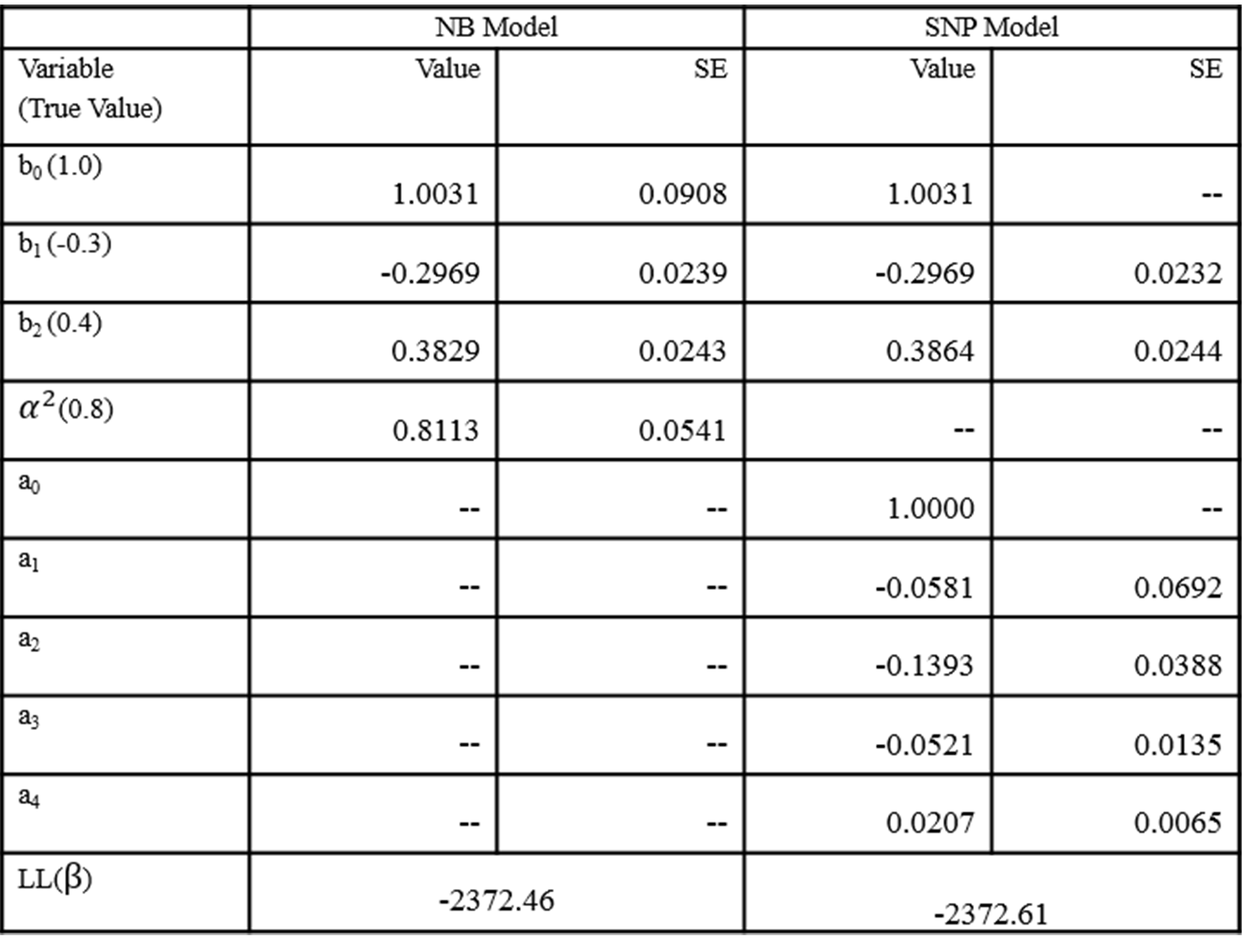

Supplement: S2 Table — (TIF) [file pone.0197338.s009.tif]

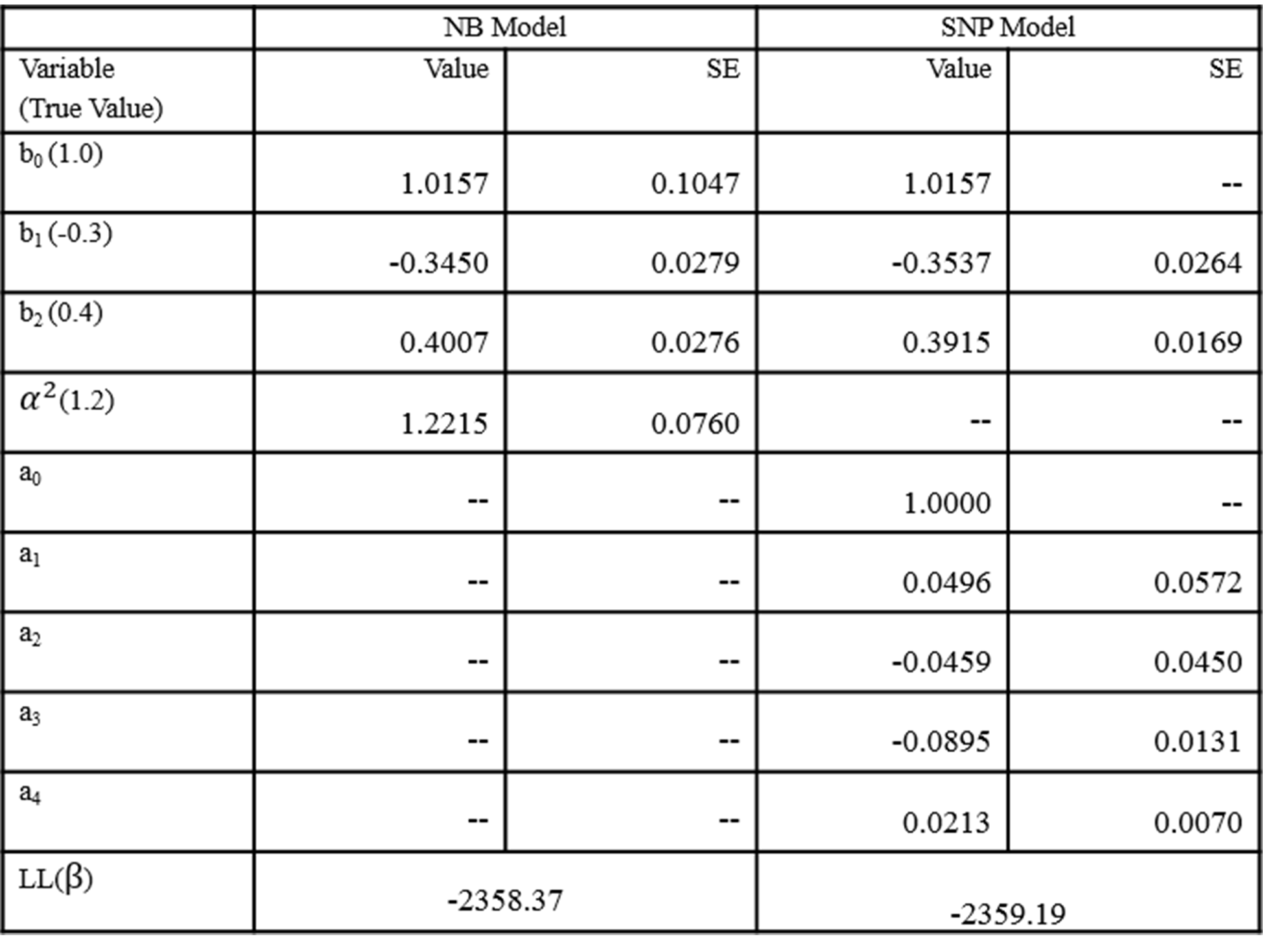

Supplement: S3 Table — (TIF) [file pone.0197338.s010.tif]

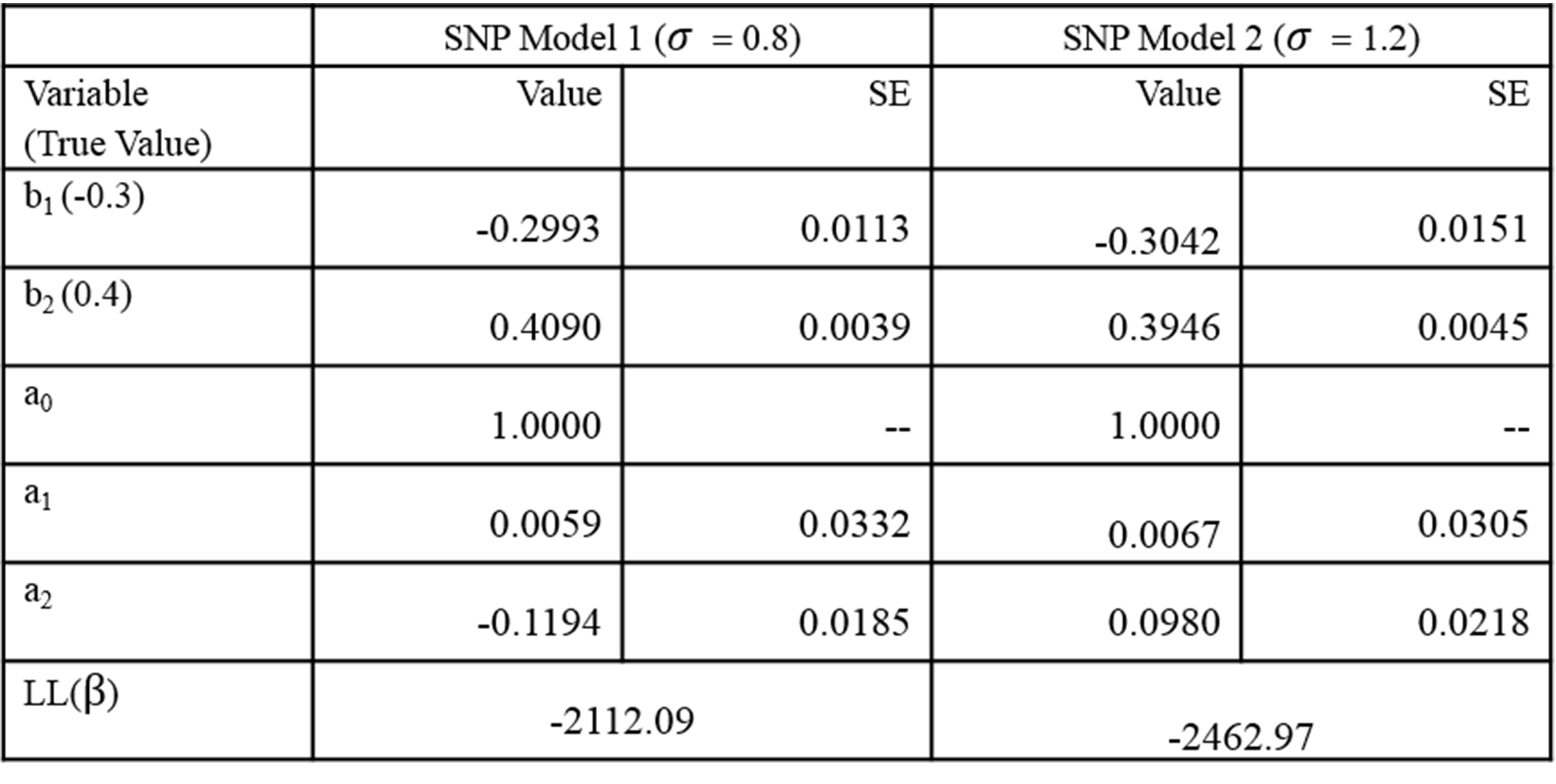

Supplement: S4 Table — (TIF) [file pone.0197338.s011.tif]

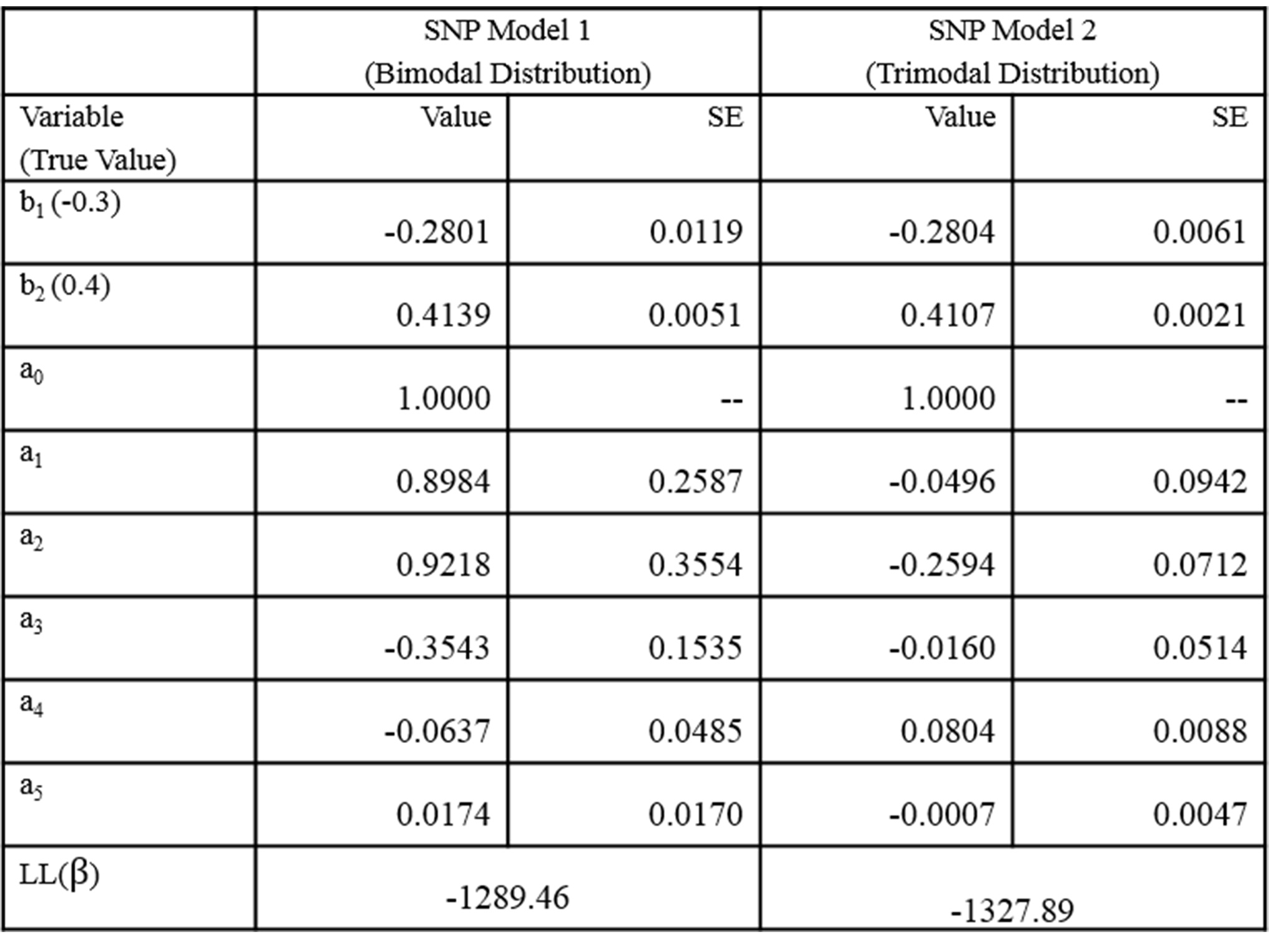

Supplement: S5 Table — (TIF) [file pone.0197338.s012.tif]

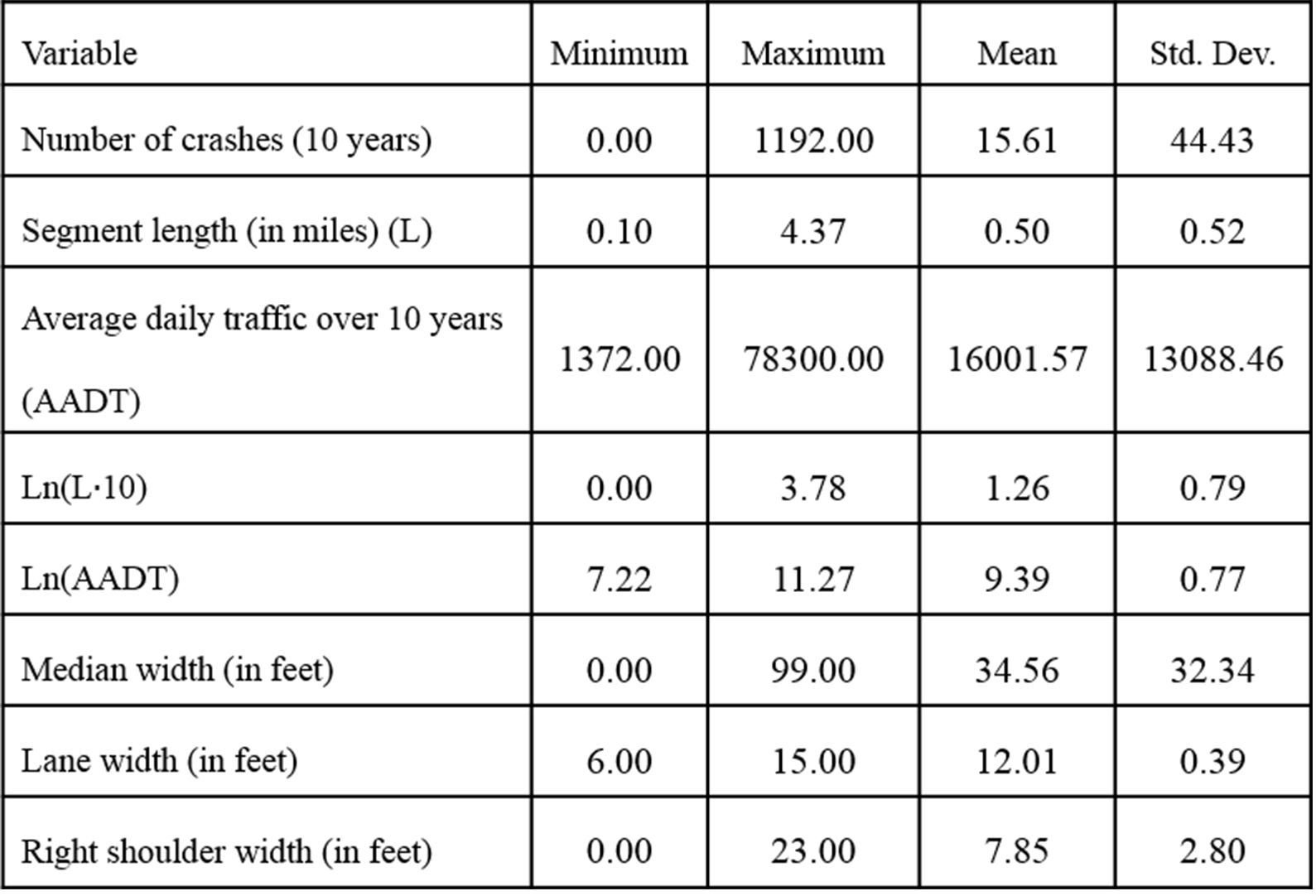

Supplement: S6 Table — (TIF) [file pone.0197338.s013.tif]

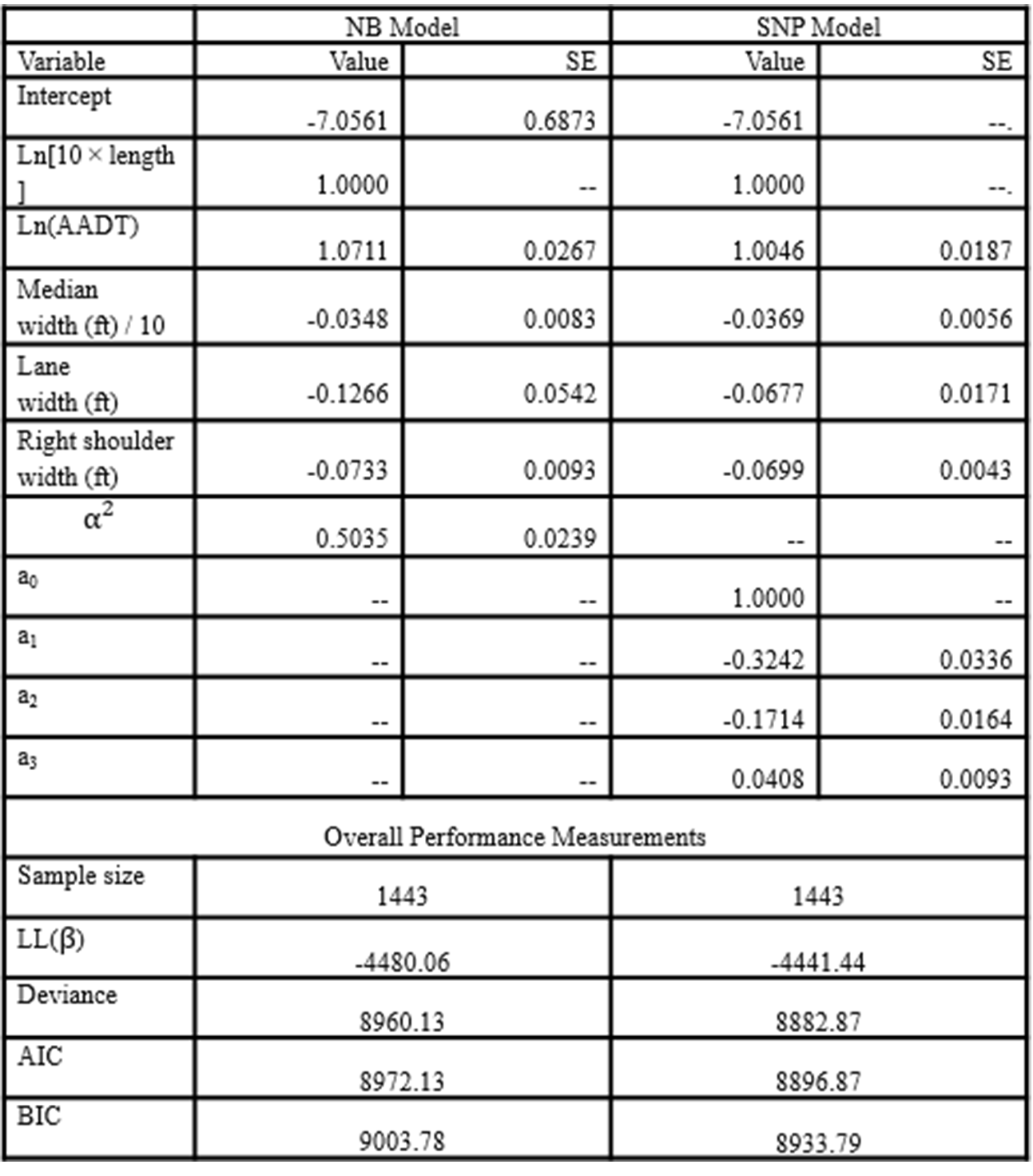

Supplement: S7 Table — (TIF) [file pone.0197338.s014.tif]
